# 330. Evaluating the Relationship Between Comorbidity Treatment Status and In-hospital Mortality Among COVID-19 Patients

**DOI:** 10.1093/ofid/ofab466.532

**Published:** 2021-12-04

**Authors:** Cristy Davenport, Sharmon P Osae, Geren Thomas, Henry N Young, Andrés F Henao Martínez, Carlos Franco-Paredes, Daniel B Chastain

**Affiliations:** 1 University of Georgia, Albany, Georgia; 2 University of Georgia College of Pharmacy, Albany, Georgia; 3 John D. Archbold Memorial Hospital, Thomasville, Georgia; 4 University of Colorado Anschutz Medical Campus, Aurora, Colorado; 5 University of Colorado Denver, School of Medicine, Aurora, CO

## Abstract

**Background:**

Chronic comorbidities increase the risk of poor outcomes in patients with COVID-19. However, there are insufficient data to determine whether control of chronic comorbidities influences outcomes. The purpose of this study was to determine whether pharmacologic treatment for common comorbidities influences in-hospital mortality.

**Methods:**

This multicenter, retrospective study included adult patients with diabetes, hypertension, and/or dyslipidemia who were hospitalized with COVID-19 in Southwest GA, U.S. Patients were divided into two groups based on treatment status, where treated was defined as documentation in the electronic medical record of outpatient pharmacologic therapy indicated for that specific comorbidity while untreated was defined as no record of pharmacologic therapy for one or more comorbidity. The primary outcome was to compare in-hospital mortality between treated and untreated COVID-19 patients. Secondary outcomes included comparing length of hospital stay, development of thrombotic events, requirement for vasopressors, mechanical ventilation, and transfer to the ICU between groups.

**Results:**

A total of 360 patients were included with a median age of 66 years (IQR 56-75). The majority were African American (83%) and female (61%) with a median Charlson Comorbidity Index of 4 (IQR 2-6). Hypertension, diabetes, and dyslipidemia were present in 91%, 55%, and 45% of patients, respectively, of which 76% (n=274) were treated. Mortality was similar between treated and untreated patients (25% vs 20%, p=0.304). Average length of stay was 9.5 days (SD 8.7) in treated patients compared to 10.6 days (SD 9.1) in untreated patients (p=0.302). No differences were observed in the rates of thrombosis (3% vs 4%, p=0.765), receipt of vasopressors (23% vs 21%, p=0.741), mechanical ventilation (31% vs 27%, p=0.450), or transfer to the ICU (27% vs 14%, p=0.112).

**Conclusion:**

Hospitalized COVID-19 patients being treated for hypertension, diabetes, and/or dyslipidemia have similar rates of complications and mortality compared to untreated patients. Further research is needed to determine whether degree of control of chronic comorbidities impacts COVID-19 outcomes.

**Disclosures:**

**All Authors**: No reported disclosures

